# First Population Study on Winter Breeding Monarch Butterflies, *Danaus plexippus* (Lepidoptera: Nymphalidae) in the Urban South Bay of San Francisco, California

**DOI:** 10.3390/insects12100946

**Published:** 2021-10-18

**Authors:** David G. James, Maria C. Schaefer, Karen Krimmer Easton, Annie Carl

**Affiliations:** 1 Department of Entomology, Washington State University, 24106 North Bunn Road, Prosser, WA 99350, USA; 2 2558 Mardell Way, Mountain View, CA 94043, USA; seasluglover@yahoo.com; 3 435 Marion Avenue, Palo Alto, CA 94306, USA; eastonfamilyfive@gmail.com; 4 2661 Waverley Street, Palo Alto, CA 94306, USA; anniejcarl@hotmail.com

**Keywords:** overwintering strategy, reproduction, non-reproductive, migration, parasite

## Abstract

**Simple Summary:**

Populations of western monarch butterflies, which formerly populated coastal overwintering sites in California in numbers exceeding one million, dwindled to less than 2000 in 2020/21. In the same winter, breeding populations of monarchs occurred commonly in the San Francisco Bay urban area for the first time. The urgent conservation need to understand this possible shift in overwintering strategy prompted this first study of the viability and ecology of monarch breeding populations in the South Bay urban area of San Francisco during January–June 2021. Adult monarchs along with eggs and larvae were common during winter and most of spring, utilizing non-native ornamental milkweed and multiple nectar sources from native and ornamental plants. Evidence from weekly counts and tagged butterflies indicated increased dispersal to the north and east during late April-mid-May, possibly representing spring migration to the Pacific Northwest and eastern California. Infection of reared adult monarchs with a protozoan parasite was high. Winter breeding of monarchs in the San Francisco urban area as an alternative and sustainable overwintering strategy for the western US population will likely depend on the continued presence of ornamental milkweeds, sustainable co-existence of monarchs and protozoan parasites, and successful migration to the greater western US during spring.

**Abstract:**

The western North American monarch butterfly population assessed by counts of non-reproductive overwintering butterflies at coastal sites in California declined to less than 2000 in 2020/21. Simultaneously, reports of reproductive monarchs increased in San Francisco urban areas, perhaps representing a shift in overwintering strategy. To better understand monarch winter breeding in the Bay area, we studied adult and immature populations in Santa Clara County during January–June 2021. Adult monarchs were common with numbers ranging from 0.23–1.54/min during ~30 min weekly surveys at one site, with lowest numbers late April to mid-May. Eggs and larvae, primarily on ornamental milkweeds, were found on nearly all survey dates with lowest numbers mid-late April to mid-May. Levels of infection of adults by the parasite *Ophryocystis elektroscirrha* were consistently high during the study (69.3–77.5%). From 499 monarchs tagged post-eclosion, recovery rates of 19.2–23.6% occurred from releases in January-February and May-June but only 11.9–13.0% from March-April releases. Although distances were small, butterflies tagged in April were recovered from greater distances than other months. Tagged monarchs flew primarily north or east. There were reduced numbers of adult monarchs during late April-mid-May with some evidence of northerly and easterly emigration at the same time from tagged butterflies, suggesting some movement out of the South Bay area, perhaps representing spring migration. We conclude that monarchs can successfully breed and maintain populations on ornamental milkweeds during winter at urban sites in the South Bay of San Francisco and may still migrate during spring to remain part of the wider western population.

## 1. Introduction

The monarch butterfly (*Danaus plexippus* (L.)) in North America is an iconic species known throughout the world for its annual return migration to southern overwintering areas in Mexico and California where it masses in trees [1]. The western population that occurs west of the Rocky Mountains is less studied than the eastern US and Canada population but both are considered to be part of a single genomic population [2]. During fall, monarchs in the Pacific Northwest as well as in inland California and the southwest US migrate to coastal California where they overwinter at more than 400 discrete overwintering sites [3,4,5]. The western population has always been much smaller than the eastern US population, but in recent years has declined dramatically, as gauged by counts at coastal overwintering sites (http://westernmonarchcount.org, accessed on: 14 October 2021). More than one million overwintering monarch butterflies were counted at California overwintering sites in 1997, but only ca.1900 were counted in 2020 [6].

Coincident with a dramatically declining overwintering population has been an apparent increase in monarch breeding activity during winter in southern California and more recently in the San Francisco Bay area [6,7,8]. This was particularly evident during the winter of 2020/21 with tagged migrants from Oregon recovered from inland urban locations with milkweed in and around San Francisco, instead of at coastal overwintering sites [6]. There was also a dramatic increase in the number of sightings of monarch larvae and pupae in the San Francisco area reported to online nature sites during winter 2020/21 compared to earlier years [6]. James [6] suggested that record warm temperatures during fall 2020 (mean daily maxima for San Francisco in September 2020 were 2.5 °C higher than the historic mean) may have caused many non-reproductive migrants flying through northern California to terminate reproductive dormancy and commence reproduction, facilitated by the recent increased availability of ornamental, non-native milkweeds grown in gardens and suburban landscapes in California.

The outcome of winter breeding of monarchs in inland California is uncertain. Whether winter breeding becomes a sink or source for monarch butterflies in the west will depend on monarch ecology in this new environment, particularly in terms of success of reproduction, survival and dispersal. Crone and Schultz [8] considered that winter breeding populations in California “probably lack the demographic capacity to expand their range inland during the summer months”. They also suggested that increased levels of the monarch protozoan parasite *Ophryocystis elektroscirrha* McLaughlin & Myers (OE) associated with year-round monarch breeding and non-native milkweeds [9], would inhibit successful spring migration. In contrast, James [6] thought that the adaptability of monarchs, especially in terms of response to environmental cues, would likely serve to ensure continuation of migration inland and northward during spring-summer, albeit perhaps at lower levels.

This study conducted in the south Bay area of San Francisco is a timely first look at the ecology of reproductive monarchs in an urban landscape during winter in northern California. We followed a winter breeding population censusing adults and immature stages, parasite infection rates and the availability of host and nectar plants. We also tagged newly eclosed monarchs to determine if increased dispersal occurred during spring which might be indicative of northward migration. If winter breeding in California is an alternative cool-season strategy for western monarch butterflies, we need data quickly so appropriate conservation strategies can be implemented.

## 2. Materials and Methods

**Figure 1:**
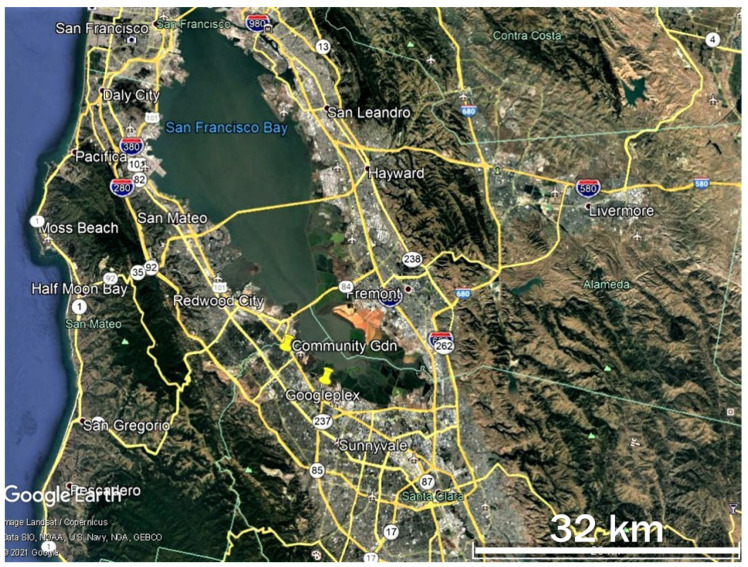
San Francisco Bay area showing locations in the south Bay (yellow pins) where this study was conducted January–May 2021 (Google Earth image).

**Figure 2:**
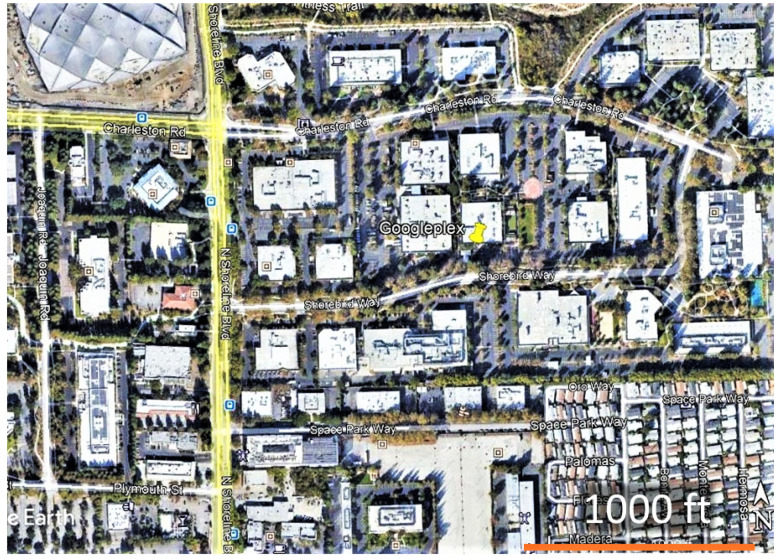
Google campus (Googleplex) at Mountain View, CA where studies on a monarch breeding population were conducted during January–May 2021 (Google Earth image).

The populations studied during January–May 2021 occurred in an ~8 by 5 km area in NW Santa Clara County, south of the San Francisco Bay, California, primarily at two sites in Mountain View (Googleplex) and Palo Alto (Rinconada Community Garden) (Figure 1). Studies on monarch breeding were conducted on a population occurring within a 0.8 by 0.3 km area, part of the Google campus at Mountain View, known as Googleplex (Figure 2). Google LLC is proactive in developing ecologically functional landscapes in and around its campuses (https://sustainability.google/progress/projects/urban-ecology/, accessed on: 14 October 2021), including creating monarch and other pollinator habitats at Googleplex and other company sites (http://blog.google/outreach-initiatives/sustainability/monarch-butterflies-california/, accessed on: 14 October 2021).

### 2.1. Adult Counts and Nectar Sources

Counts of adult monarchs commenced on 31 January at Googleplex and continued weekly until 31 May 2021. Counts were conducted by one of us (M.C.S.) when conditions were sunny and the temperature was at least 14.4 °C at the beginning of the count. Temperatures during the count were usually 15–25 °C and wind conditions varied from calm to breezy. Butterflies were sighted and counted during an established walking route along landscaped roads (primarily Shorebird way and Charleston Road) with abundant milkweed (primarily ornamental Tropical milkweed, *Asclepias curassavica* L.) that took 24–28 min to complete. Available nectar sources were noted on each count. Casual observations were also made on butterfly behavior and condition. Nectar sources and butterfly behavior were also recorded on frequent visits during January–June to Rinconada Community Garden.

### 2.2. Counts of Eggs and Larvae

Counts of eggs and larvae commenced on 2 February and continued weekly until 31 May 2021. *Asclepias curassavica* was the dominant milkweed species at Googleplex with smaller numbers of native milkweeds, *Asclepias speciosa* Torr. (Showy milkweed) and *Asclepias fascicularis* Dcne. (Narrow-leaved milkweed). Primary counts were made on 10 randomly selected *A. curassavica* plants that each occupied an area of at least 0.6 m^2^ and were taller than ~0.3 m.

An estimated 60 plants of this size or greater existed on the route used for adult counts and ten were randomly selected (using a random number generator) on each survey and thoroughly examined using a magnifying glass (when necessary) for eggs and larvae. All leaves and flowers were searched and larvae were assigned to instar based on length and other characteristics (https://www.youtube.com/watch?v=IuPsK6xOnDY&t=319s, accessed on: 14 October 2021). From mid-April until the end of May, counts were also made of eggs and larvae on three randomly selected *A. speciosa* (from 11) and three *A. fascicularis* (from six) plants, located on the same survey route. A lack of leaves on these native milkweeds prevented earlier counts on these plants. Although not specifically searched for, a small number of monarch pupae were found on *A. curassavica* and adjacent plants. The outcome of these pupae was recorded.

### 2.3. Infection of Monarch Adults by the Protozoan Parasite Ophryocystis Elektroscirrha (OE)

During January–May 2021, mid-late instar larvae were collected from non-surveyed plants at Googleplex and also from the Rinconada community garden/monarch waystation located in Palo Alto. In the latter location, eggs and early instar larvae were also collected. Collected eggs and larvae were reared on milkweed (primarily locally obtained *A. curassavica* and *A. fascicularis*) under protected conditions, primarily outdoors in a greenhouse or indoors with exposure to ambient daylengths and temperatures ranging from 15–25 °C. Newly eclosed adults were sexed and tested for OE using the tape count method, in which sticky tape is pressed against the abdomen and then placed on a white card for later examination under a stereomicroscope [10]. Spores of OE were counted or estimated (depending on density) on a 1 × 1 cm section of sticky tape. Spore density was graded as follows: 1 = no spores, 2 = <100 spores, 3 = 100–1000 spores and 4 = >1000 spores. Grades 1 and 2 are considered ‘uninfected’ since infections of <100 spores could be the result of contamination [10].

### 2.4. Dispersal and Movement of Adult Monarchs

Reared monarchs were tagged with a single tag placed on the discal cell on the ventral surface of a hindwing. Tags were obtained from MonarchWatch.org and customized with a serial number and a Washington State University email address (monarch@wsu.edu) for contact. For all tagged monarchs, the date of tagging/release, location of release, tag number and sex were recorded. Forewing length (mm) was also measured. All tagged monarchs were released during January–June in the Redwood City-Palo Alto-Mountain View area, close to where they were sourced as immatures. During February, ~60 tagged butterflies were released at Googleplex. Tagged monarchs were primarily recovered by citizens sighting or photographing live butterflies. Occasionally, deceased butterflies were found and reported. Data obtained from recovered butterflies included monthly recovery rates, elapsed period between release and recovery and distance/direction flown.

Statistical analyses were conducted using SigmaStat Version 3.0 SPSS Inc., (Chicago, IL, USA) software. To compare monthly tag recovery rates and recoveries from distances >1.0 km, we used a simple chi-square contingency table analysis. Monthly data on forewing length, OE ratings, period between release and recovery of tagged butterflies and distances flown by tagged butterflies were analyzed using analysis of variance (ANOVA). Data were log (log x) transformed prior to analysis by ANOVA to improve normality of variances and then back-transformed for reporting.

## 3. Results

**Figure 3:**
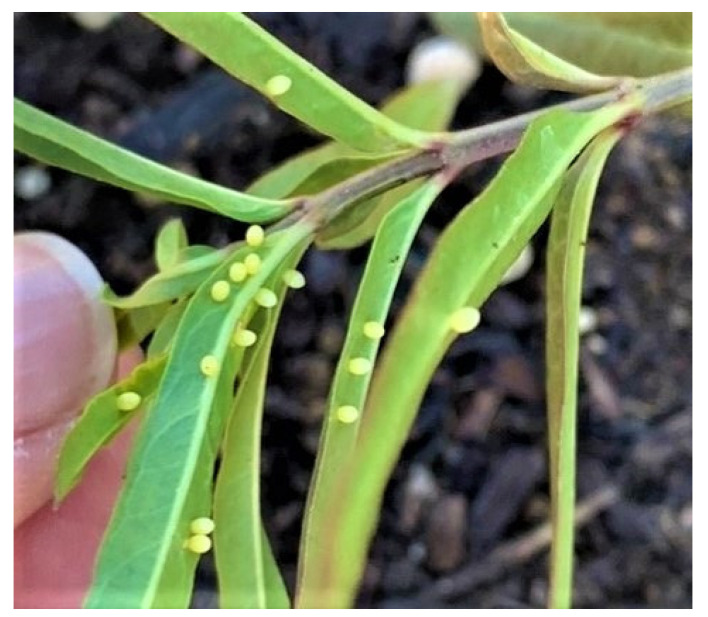
High-density oviposition by monarchs on *Asclepias fascicularis* at Rinconada Community Garden, Palo Alto, CA, USA, 23 February 2021.

Monarch butterflies occurred commonly throughout the Palo Alto-Mountain View area prior to (November-December) and during this study. High population densities occurred at the Rinconada Community Garden and at Googleplex with dozens of butterflies flying at these locations on sunny days during November 2020–January 2021. Temperatures during January–May ranged from 1 °C (26 January) to 31.1 °C (10 May) with monthly mean maxima ranging from 16.3 ± 1.1 °C (January) to 21.4 ± 0.7 °C (May) and minima, 6.7 ± 0.3 °C (March) to 11.3 ± 0.3 °C (May) (Moffett Field, Mountain View, http://timeanddate.com, accessed on: 14 October 2021). In addition, sunshine occurred on nearly every day with only three entirely overcast days between 15 January and 31 May. At Rinconada Community Garden, evidence of high-density oviposition on limited numbers of milkweed (primarily *A. curassavica, A. fascicularis, Gomphocarpus physocarpus,* E. Mey, *Gomphocarpus fruticosus* (L.) W. T. Aiton) was observed (Figure 3).

### 3.1. Adult Counts and Nectar Sources

**Figure 4:**
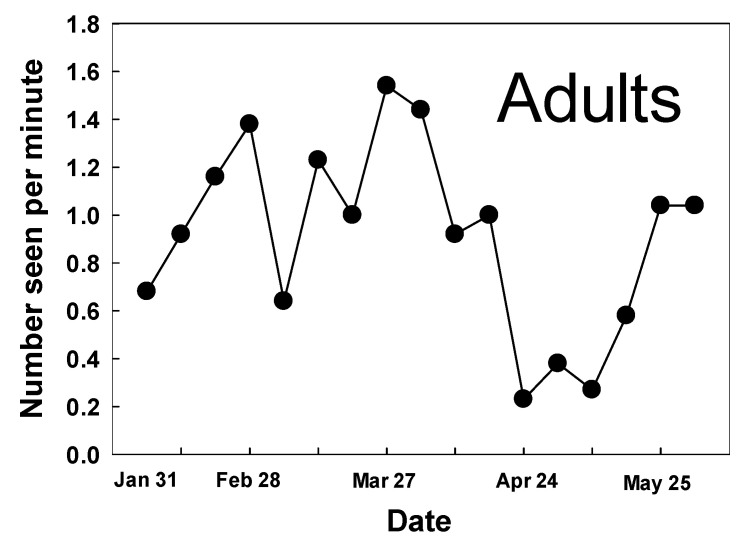
Monarch adults recorded during weekly surveys at Googleplex, Mountain View, CA, USA, 31 January–31 May 2021.

Monarchs were recorded flying and active on all 17 weekly counts conducted between 31 January and 31 May (Figure 4). Total numbers ranged from 6 on 24 April to 40 on 27 March with mean number per minute ranging from 0.23 to 1.54 (Figure 4). The population generally increased from January until the end of March when it declined from 1.54/min to 0.23/min over four weeks. From 23 April to 18 May, numbers remained well below 1.0/minute, before climbing on the last two survey dates in May.

Twenty-one native and ornamental plant species present at Googleplex bloomed during winter and spring, providing nectar for adult monarchs (Table 1). Nine species bloomed during winter (December–February) and monarchs were seen feeding on these species and most of the others that bloomed during spring. The primary host plant for monarchs at Googleplex, *A. curasssavica,* remained green and suitable for oviposition and larval development throughout the winter; however, it did not begin blooming until the middle of May (Table 1). Only three ornamental nectar sources occurred within Rinconada Community Garden flowering throughout the winter (Euryops, *Euryops pectinatus* (L.) Cass., Rosemary, *Salvia rosmarinus* Spenn. and Field mustard, *Brassica rapa* L.). All were used by monarchs and we suspect additional nectar sources in nearby gardens were also utilized.

### 3.2. Counts of Eggs and Larvae

**Figure 5:**
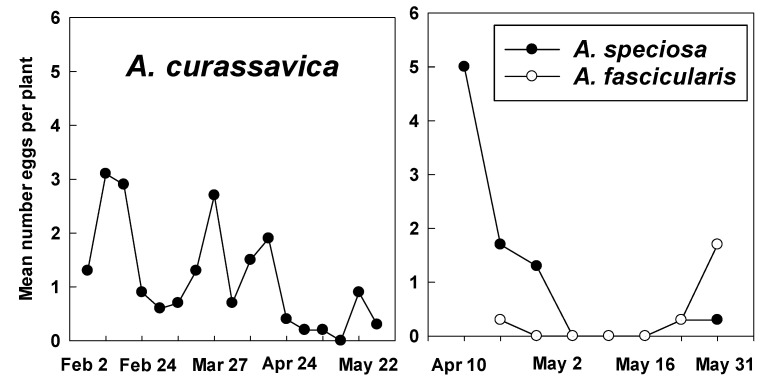
Monarch eggs recorded weekly on tropical milkweed (*A. curassavica*) (2 February–31 May) and native milkweeds, *A. speciosa* and *A. fascicularis* (10 April–31 May) at Googleplex, Mountain View, CA, USA.

Eggs were recorded on *A. curassavica* on all dates except 16 May, with greatest numbers in mid-February (2.9–3.1 eggs/plant) and briefly at the end of March (2.7 eggs/plant). Lowest numbers occurred during 24 April–16 May (0–0.4 eggs/plant) (Figure 5). Higher numbers of eggs on *A. speciosa* and *A. fascicularis* also showed a steep decline from mid-April to mid-May (Figure 5).

**Figure 6:**
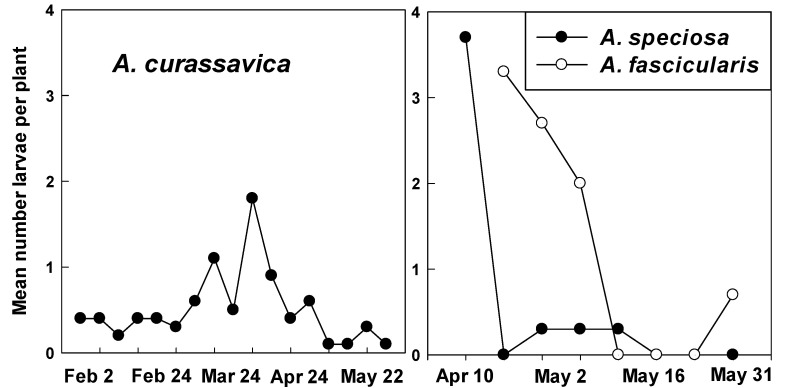
Monarch larvae recorded weekly on tropical milkweed (*A. curassavica*) (2 February–31 May) and native milkweeds, *A. speciosa* and *A. fascicularis* (10 April–31 May) at Googleplex, Mountain View, CA, USA.

Larvae were found on *A. curassavica* on all dates ranging from 0.1–1.8/plant. Higher numbers (0.5–1.8/plant) occurred from 27 March to 18 April with lower numbers present during late April to mid-May (Figure 6). Higher numbers of larvae on *A. speciosa* and *A. fascicularis* also declined during mid-April to mid-May (Figure 6).

**Figure 7:**
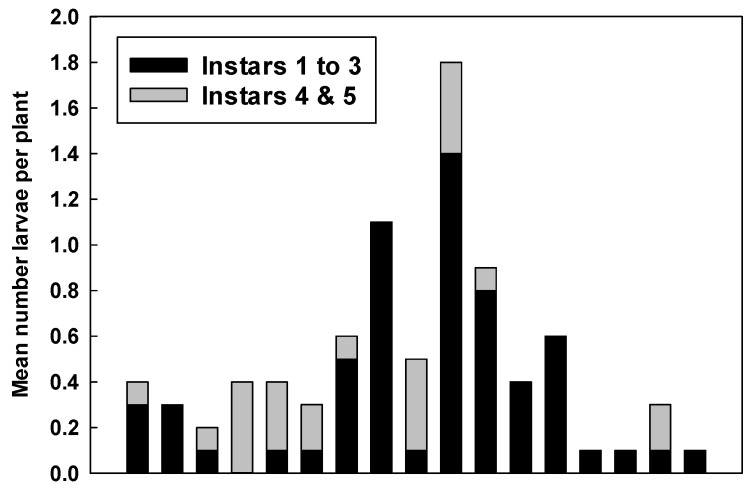
Numbers of young (instars 1–3) and mature (instars 4–5) monarch larvae recorded weekly on tropical milkweed (*A. curassavica*) at Googleplex, Mountain View, CA during 2 February to 31 May 2021.

Separating larvae into early instars (1–3) and late instars (4–5) indicated that early instars dominated the counts (Figure 7) and were responsible for the peaks seen in overall numbers of larvae. Mature larvae were most common in early March and early April.

Seven pupae were found on *A. curassavica* (4), adjacent grasses (2) and *L. perezii* (1) during 31 December and 2 February. Of these, two produced adults in mid-late February, 25 and 34 days after being found. The remainder disappeared (3) or were non-viable (2).

### 3.3. Infection of Monarch Adults by the Protozoan Parasite Ophryocystis elektroscirrha (OE)

Monarchs (*n* = 492) that eclosed between 17 January and 12 June were tested for the presence of OE spores. Of these, 356 (72.4%) had 100 or more spores and 46.3% (228) were heavily infected with >1000 spores. Only 136 (27.6%) had zero or less than 100 spores. Periodic evaluation of infection level from January/February to May-June indicated little difference, with infection levels ranging from 69.3 to 77.5% (Table 2). Similarly, mean OE ratings varied from 2.90 ± 0.098 to 3.13 ± 0.110 during January to June and did not differ significantly between months (ANOVA, *F* = 0.841, df 3, 487; *p* = 0.472) (Table 2).

### 3.4. Dispersal and Movement of Adult Monarchs

From 29 January to 12 June 2021, 499 newly eclosed monarchs were tagged and released. Mean forewing length was 47.8 ± 0.15 mm and monthly means did not differ significantly ranging from 47.3 ± 0.29 mm (April) to 48.4 ± 0.49 mm (Jan/Feb) (ANOVA, *F* = 1.544, df 3, 367; *p* = 0.203). Of these, 84 (16.8%) were recovered at least once. The number released each month varied from 108–134 and recoveries ranged from a high of 25 and 30 (19.2–23.6%) from January–February and May–June releases, to a low of 14–15 (11.9–13.0%) from March and April releases (Table 3). The mean period between release and recovery of tagged butterflies was 22.6 ± 1.9 days overall. This period was slightly longer for butterflies tagged and released in January-February (27.8 ± 4.4 days) but not significantly so (ANOVA, *F* = 0.186, df 3, 74; *p* = 0.906) (Table 3).

**Figure 8:**
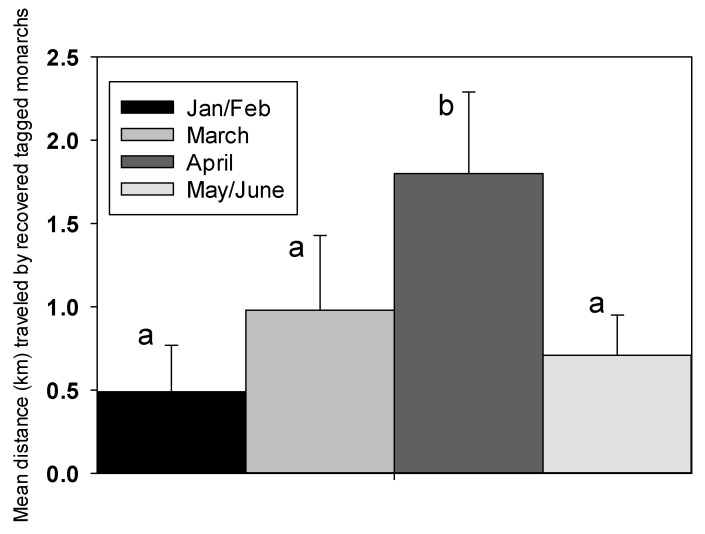
Mean (±SE) distances (km) flown by recovered tagged monarch butterflies in Santa Clara County, CA during January–June 2021. Column denoted by a different letter is significantly different (*p* < 0.05).

Distances flown by tagged monarchs were relatively small (0–6.4 km). However, butterflies tagged in April were recovered from significantly greater distances than those tagged in other months (ANOVA, *F* = 2.39, df 3, 78; *p* = 0.044) (Figure 8).

The cardinal and ordinal directions flown by recovered tagged monarchs were primarily N, NW and NE in each month of release. Most of the remainder flew SE-ESE. From 26 butterflies that moved at least 1.0 km during January–June, 19 (73.1%) were recovered NW-N-NE of release points. The remainder were found ESE-SE of release points, with only one recovery west (WSW) of the release point.

## 4. Discussion

This study arose following online and social media investigations by DGJ in January 2021 on the extent of winter breeding by monarchs in urban areas of the San Francisco Bay area [6]. James [6] considered the rise in winter breeding monarch populations in California in 2020/21 was likely a result of fall migrants becoming reproductive due to increased autumn temperatures and the widespread availability of ornamental non-native milkweeds. James [6] also recognized that the function and outcome of winter breeding was uncertain in terms of maintenance of the larger western US population, because of the possibility that winter resident monarchs may not produce spring migrants to colonize the greater west [8]. Consequently, the importance of having timely data on the apparent widespread winter breeding urban populations in the Bay area in 2020/21 prompted this initial study. These data provide a ‘first look’ at the winter ecology of urban monarchs in the San Francisco region.

### 4.1. Successful Winter Breeding

**Figure 9:**
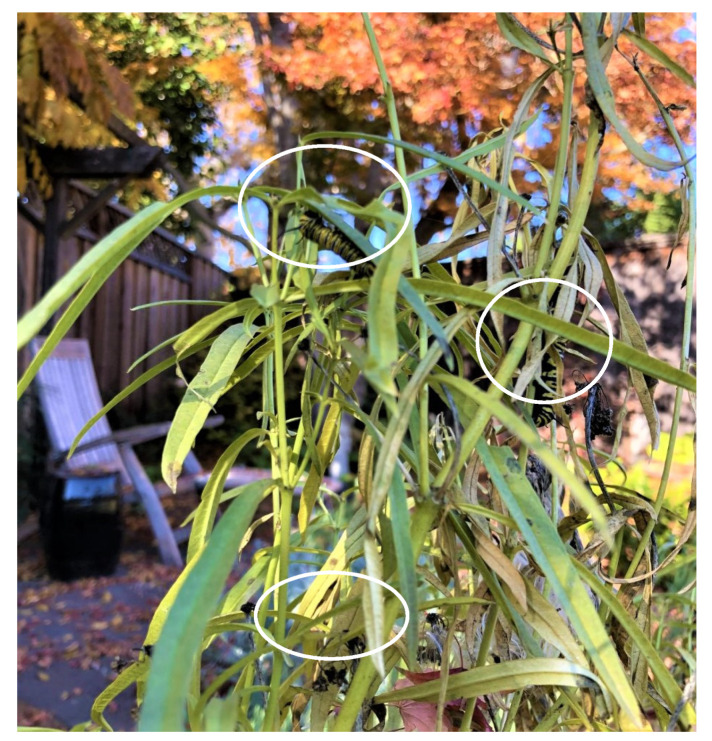
Native milkweed, *Asclepias fascicularis,* and late instar monarch larvae (circled) in a Palo Alto backyard on 3 December 2020 (Photo: John Tang).

Monarch butterflies were common in urban areas of NW Santa Clara County, CA throughout winter 2021/21. Monarchs were frequently seen in community gardens, home gardens and parks, particularly where milkweeds and/or flowering plants were present. Our observations at Rinconada Community Garden, Palo Alto and Googleplex at Mountain View revealed substantial numbers of adult monarchs during January–March. At the community garden where milkweed was limited, numerous instances of ‘egg-dumping’ by female monarchs were recorded (Figure 3, see also Figure 3 in James [6]). The non-native Tropical milkweed (*A. curassavica*), Balloon milkweed (*G. physocarpus*) and Swan plant (*G. fruticosus*) remained in a largely suitable condition for oviposition and larval development through the winter. In contrast, the native milkweeds, *A. speciosa* and *A. fascicularis*, normally die back in late fall and are not available for oviposition until early spring. However, we found evidence in 2020/21 that *A. speciosa* and *A. fascicularis* remained green longer during fall in the south Bay area than was expected. In December 2020, there were still reports of monarch larvae on non-senesced *A. fascicularis* in Palo Alto gardens (Figure 9).

Evidence of sustained reproductive activity occurred throughout the study as shown by the continual presence of eggs and larvae. Monarch pupae are uncommonly found in the wild but seven were found during January with two, after extended durations, producing adults. The adult population at Googleplex was reproductive during the period of study with eggs found on all survey dates except one. Generations as distinguished by bursts of fresh adults were difficult to discern and appeared to be relatively unsynchronized, which is characteristic of monarch breeding populations in the western US [11]. However, peaks of oviposition occurred in mid-February and at the end of March, likely representing major periods of new adult emergence. Releases of ~60 reared monarchs at Googleplex during February may have contributed to the increase seen during that month. The greatest numbers of adults occurred at the end of March. Thereafter, adult, egg and larval numbers declined to the lowest levels of the study with the period from 20 April to 20 May, particularly characterized by low numbers of all monarch life stages on all three species of milkweed monitored at this time. The paucity of adults flying at Googleplex was particularly marked. Late April-late May is the usual time frame for emergence of the first locally-produced new generation of monarchs in northern California, resulting from oviposition in late February and March by females from coastal overwintering colonies [12]. These progeny from overwintering monarchs migrate northward from late April to early June, populating Oregon, Washington and Idaho [4]. We speculate that the relative absence of adult monarchs and lower numbers of eggs and larvae at Googleplex during April-May may have been a consequence of the local adult population showing increased dispersal northwards and eastwards, effectively joining the spring migration.

### 4.2. Spring Dispersal or Migration

While no long-distance recoveries were made of monarchs we tagged during February to June, data on monthly recovery rates, period between release and recovery and distance flown suggest there was a period between March and June when butterflies were more dispersive. A high recovery rate of tagged monarchs may indicate little dispersal and this occurred in January (19.2%) and May–June (23.6%). Recovery rates in March and April were lower (11.9–13.0%) and may indicate greater dispersal, although this difference was not statistically significant. A reduced period between release and recovery during March–June compared to February may represent reduced longevity related to higher temperatures, but there may have also been dispersal of longer-lived migratory individuals. Although distances flown by tagged butterflies in our study were small, they were significantly greater in April than any other month. The majority (73.1%) of recovered tagged monarchs that flew >1.0 km, were found north of release points (NW-N-NE), while the remainder, except one (WSW), were found east (ESE-SE) of release points. Release points for our tagged monarchs were surrounded fairly evenly on all sides by comparable human population density, making it just as likely for a tagged butterfly to be found to the south or west as to the north and east. Spring migrants in California are known to have northerly or easterly trajectories [12]. If migration away from the South Bay area occurred during April–May, why did we not have recoveries of tagged monarchs from further afield? The most compelling evidence for a movement of adult monarchs away from the South Bay area comes from the survey data at Googleplex showing substantially reduced numbers between 24 April and 7 May. Only 53 monarchs were tagged during this period, which is not enough to expect a long-distance tag recovery. Less than 1% of fall-tagged monarchs were recovered at long distances in migration studies on the western monarch population [4,5]). Even if all monarchs tagged during April–June (235) are considered potential migrants, only two or three long-distance recoveries might be expected. Consequently, we believe we did not tag enough butterflies during the most likely migration window for the South Bay population (late April to late May) to have a good chance of obtaining long-distance tag recoveries.

### 4.3. Infection by O. elektroscirrha (OE)

Infection by the protozoan parasite *O. elektroscirrha* (OE) was common in newly eclosed adult monarchs throughout this study. Little more than a quarter of the monarchs we tested were free of or had fewer than 100 OE spores, and infection levels of 69–77% were consistent during winter and spring. These infection levels may have been exacerbated a little by captive rearing which may have resulted in some transmission among larvae reared together. However, most transmission of OE occurs via females contaminating newly laid eggs with spores, and thus our recorded infection levels are likely close to the natural levels. In a study on year-round breeding populations of monarchs on *A. curassavica* in southern California, Satterfield et al. [9] reported a comparable prevailing OE infection rate of 74%. An earlier study [13] found that monarchs at two overwintering sites in California were also infected with OE at rates between 53 and 68%, although Satterfield et al. [9] reported only 8% prevalence of OE at eight overwintering sites. OE infection has the potential to be lethal or debilitating for monarchs by reducing eclosion success, and causing wing deformities, shortened adult life span and reduced flight performance [14,15]. However, most of the studies to date on the impact of OE on monarch health have been conducted on the eastern US population of *D. plexippus*. There is limited evidence that impacts may be less severe on monarchs in the western US, with a recent study showing no apparent difference in longevity and migration ability between OE-infected and uninfected butterflies [5]. In the current study, none of the 228 butterflies that were heavily infected with >1000 spores each had wing deformities. Nevertheless, it is possible that high levels of OE infection in winter-breeding monarchs in the Bay area could compromise the ability of the population to migrate northwards in spring [6,8], and this needs to be investigated urgently.

### 4.4. Role of Non-Native Milkweeds in Winter Breeding

This study on monarch butterflies in the South Bay area of San Francisco during winter confirms that good survival and successful reproduction can occur if milkweed host plants are available. Temperatures during January–June 2021 in Santa Clara County (Moffett Field, Mountain View, http://timeanddate.com, accessed on: 14 October 2021) were favorable for monarch breeding, with daily maxima averaging 18.2 °C with abundant sunshine and no sub-freezing nights. The long-term (1985–2015) mean maxima for January-June at this site is also 18.2 °C, although the mean daily maxima for January and February 2021 were 1.8 and 0.9 °C higher than the long-term means. The impact of non-native tropical milkweeds on monarch ecology in the US is controversial with some studies associating monarch use of these plants with negative impacts including increased OE prevalence [9] and possible disruption of migration [16]. However, there seems to be little doubt that without the presence and viability of *A. curassavica, G. physocarpus* and *G. fruticosus* in the South Bay area during winter 2020/21, sustained breeding populations of monarch butterflies would not have existed at the same density. Although native milkweeds remained viable in some locations into December, they senesced and were not available during January–March. If non-senesced non-native milkweeds had not been available in 2020/21, what would the outcome have been for breeding populations of monarchs in NW Santa Clara County on native milkweeds? Adults would have needed to ‘bridge the gap’ during January-April when native milkweeds were fully senesced. While survival of reproductive adults during this cool period is possible, it would likely have resulted in a serious population crash. If there are negative impacts of non-native milkweeds on the biology and ecology of monarchs, clearly none could be as dire to the population as milkweed absence. Public confusion surrounding the impact of non-native milkweeds on monarch ecology and health was highlighted during this study when we observed that *G. physocarpus* plants with multiple monarch eggs and larvae were ripped up during December at one location and composted. This was done because of perceived ‘negative effects’ of non-native milkweeds on monarchs. While *G. physocarpus* and *G. fruticosus* are old world milkweeds from Africa introduced into North America by man, *A. curassavica* originated much closer to the US. Woodson [17] considered its origin to be conjectural but most likely to be central America, the Antilles, Mexico or southern South America. The range of *A. curassavica* encompasses all these areas and with a warming climate, the natural range of *A. curassavica* likely now includes far southern US even if it did not previously. Continuously breeding monarch populations occur in many of the possible origin areas of *A. curassavica,* so it could well be an ancestral host plant of the monarch. Thus, *A. curassavica* may not be ‘non-native’ as far as an association with the monarch is concerned.

### 4.5. Extent of Winter-Breeding in California

While the winter of 2020/21 appears to have been the first in which substantial winter breeding of monarchs has occurred in the San Francisco Bay area [6], winter breeding has been reported and known from the Los Angeles Basin since 1970 [18]. During 2013–2016, Satterfield et al. [9] sampled monarchs at 42 year-round breeding sites in southern California. It is difficult to know whether winter breeding has further increased in southern California in recent years, but reports on online social media indicated substantial populations occurred during 2020/21. One Facebook page with less than 650 members reported 2361 sightings of monarch life stages in southern California during March–May 2021 (Susie Vanderlip, pers. comm.). Whether winter breeding populations occurred in other near-coastal urban areas between Los Angeles and San Francisco in 2020/21 is uncertain, but may have occurred wherever non-native milkweeds were cultivated. Also uncertain was the extent and size of breeding populations in comparison to non-breeding populations during winter 2020–21. While only ~1900 butterflies were recorded from coastal overwintering sites, it is possible this number may have been dwarfed by numbers in breeding populations that occurred in the San Francisco Bay area, southern California and possibly locations in-between. Clearly, unlike non-breeding populations clustering at discrete overwintering sites, it is difficult to estimate the size of winter breeding monarch populations of monarchs scattered over a wide area of the state.

### 4.6. Winter Breeding in the Bay Area May Contribute to the Western Population

Although this study has shown convincingly that it is possible for monarchs to overwinter and reproduce in urban areas of the South Bay of San Francisco, it may not dispel the fear expressed by others (e.g., Crone and Schultz [8]) that a resident winter breeding population in California may not effectively contribute to spring migration, thus creating a western US population that is largely restricted to urban areas in California. Although our evidence for spring dispersal and migration from an urban resident monarch population is largely circumstantial, it appears to be real. Additional circumstantial evidence for possible migration north from reproductive populations comes from reports of migrant monarchs in the Pacific Northwest during summer 2021. During June and July 2021, 31 monarchs were reported in Washington and Oregon. This is slightly more than the number (26) reported during the same period in 2020, which followed an overwintering count of 29,436 (http://westernmonarchcount.org, accessed on: 14 October 2021; June–July numbers in 2020 and 2021 were compiled from http://journeynorth.org, http://i-naturalist.org, accessed on: 14 October 2021, monarch Facebook sites and personal reporting to DGJ). The slightly greater numbers seen in summer 2021 after an overwintering count that was <7.0% of that recorded in 2020, suggests that many monarchs migrating north in 2021 may have originated from sources other than populations at overwintering sites. However, definitive proof of a spring migration in California that includes individuals from resident populations in urban areas must await further study, which should include large-scale tagging of spring monarchs from these areas. Similarly, the possible impact of OE infection on the success of migrants colonizing the Pacific Northwest and eastern parts of California must also be examined and characterized.

### 4.7. Winter Breeding and Climate Warming

This first study on winter breeding monarchs in the San Francisco Bay area has confirmed the anecdotal reports of increased numbers of larvae in the area referenced by James [6] and Crone and Schultz [8] and shown that reproduction on non-native ornamental milkweeds can occur during January–May. Small numbers of larvae have been reported during winter in the San Francisco area for at least five years but the winter of 2020/21 was the first in which substantial numbers were reported [6]. Whether this is the beginning of a new period of substantial winter breeding in urban areas of San Francisco, or an oddity caused by record high autumn temperatures in 2020 [6] remains to be seen. It is possible that seasonal autumn temperatures in future years in northern California may see a return to minimal winter breeding in San Francisco and larger colonies at coastal overwintering sites. However, autumns that are characterized by well above average temperatures may result in larger populations of winter breeding monarchs than non-reproductive, overwintering monarchs, as appeared to be the case in 2020/21. This latter scenario appears more likely given current global warming trends. The importance of ornamental, non-native milkweeds to the current success of monarch winter breeding in urban areas of San Francisco cannot be overstated.

### 4.8. Dependence of Winter Breeding on Non-Native Milkweed

Although native milkweeds responding to a warming climate appear to be staying green longer, there is still a period between January and April when they are currently not available. Maintaining green cover of non-native milkweeds during winter for survival of breeding monarch populations is at odds with current recommendations by monarch conservation organizations to cut back these milkweeds during fall and winter (https://monarchjointventure.org/images/uploads/documents/Oe_fact_sheet.pdf, accessed on: 14 October 2021). This recommendation is based on a strategy to break the OE infection cycle. However, if a good proportion of the western monarch population is dependent for its survival on the presence of green non-native milkweeds during winter, then this recommendation needs to be reconsidered for California. More research is urgently needed on the effects and impact of OE on western monarch populations specifically.

### 4.9. Monarchs Adapting to a Changing Environment

The existence of breeding and non-breeding monarch butterfly populations in a single geographic region is not unprecedented. Non-reproductive colonies formed by migrants and reproductive populations of monarchs co-exist during winter in the Sydney Basin, New South Wales, Australia [19]. First described in the late 1970s [20,21], winter breeding and non-breeding monarch populations still exist, sometimes side by side, in the Sydney Basin [22]. Importantly, monarch migration still exists in New South Wales, with migrants forming non-reproductive overwintering colonies. There is no reason why this scenario of continued fall migration and co-existence of winter reproductive and non-reproductive monarch populations in California should not become the ‘new normal’ for western North American monarchs. This would be one more example of the amazing adaptability of this iconic insect.

## Figures and Tables

**Table 1:** Nectar plants used by monarchs at Googleplex and blooming periods.

Plant Species	Blooming at Googleplex 2021	Blooming Period: Santa Clara County	Native or Ornamental
*Limonium perezii* (Stapf)	31 Jan–31 May	Year-round	Ornamental
*Verbena lilicina* Greene	31 Jan–31 May	Year-round	Ornamental
*Lantana camara* L.	31 Jan–31 May	Year-round	Ornamental
*Gaillardia aristata* Pursh	31 Jan–31 May	Year-round	Native
*Achillea millefolium* L.	31 Jan–31 May	Year-round	Native
*Erigeron glaucus* Ker Gawl	31 Jan–31 May	Year-round	Native
*Lobularia maritima* (L.) Desv.	31 Jan–31 May	Year-round	Ornamental
*Arctostaphylos* sp.	31 Jan–27 Mar	Winter	Native
*Ribes malvaceum* Sm.	31 Jan–11 April	Winter	Native
*Lupinus* spp.	31 Jan–18 April	Spring	Ornamental
*Rhus ovatra* S. Watson	31 Jan–31 Mar	Winter–spring	Native
*Solidago californica* Nutt.	31 Jan–2 April	Summer–fall	Native
*Symphotrichum chilense* (Nees) G.L Nesom	31 Jan–21 Feb	Summer–fall	Native
*Frangula californica*(Eschsch) A. Gray	21 Feb–21 Mar 16–31 May	Spring–summer	Native
*Ceanothus* spp.	21 Feb–24 April	Spring	Native
*Salvia sonomensis* Greene	6 March–7 May	Spring–summer	Native
*Echium candicans* L.f.	27 March–30 April	Spring–summer	Ornamental
*Lupinus albifrons* Benth	27 March–24 April	Spring–Summer	Native
*Nepeta faasenii* Bergmans ex Stearn	11 Apr–31 May	Spring–summer	Ornamental
*Asclepias fascicularis* Dcne	16–31 May	Spring–fall	Ornamental
*Monardella villosa* Benth	31 May	Spring–summer	Native

**Table 2:** Mean monthly OE infection percentage and OE rating of adult monarchs reared from larvae collected in Santa Clara County, CA during January–June 2021. OE Rating key: 1 = zero spores, 2 = <100 spores, 3 = 100–1000 spores, 4 = >1000 spores. No significant difference in ratings between months (*p* = 0.472).

Months	Jan–Feb	March	April	May–June
% infected (>100 spores)	73.0	69.3	70.1	77.5
Mean (±SE) OE Rating	3.05 (0.097)	2.90 (0.098)	2.92 (0.111)	3.13 (0.110)
No. examined	137	137	107	111

**Table 3:** Tag and recovery data for monarchs tagged in Santa Clara County during January–June 2021. Statistical analysis shows that differences in recovery rates between months were minor. (Chi-square test, 2 × 4 contingency table, Chi-square = 2.70, df = 3, *p* = 0.439). Release-recovery periods (days) of tagged monarchs was not significantly different between months (*p* > 0.05).

Months	Jan–Feb	March	April	May–June
No. Tagged	130	134	108	127
No. Recovered	25	15	14	30
% Recovered	19.2	11.9	13.0	23.6
Mean (±SE) period (days) from release to recovery	27.8 (4.4)	19.3 (3.3)	21.1 (4.3)	20.6 (2.9)
